# Hormone Receptor Down-Regulation in Metastatic Breast Cancer After Endocrine Therapy Detected in Oophorectomy: A Case Report and Review of Literature

**DOI:** 10.7759/cureus.19994

**Published:** 2021-11-29

**Authors:** Marina Barron, Amira Asaad, Philip Idaewor, Noreen Rasheed, Abdalla Saad Abdalla Al-Zawi

**Affiliations:** 1 Emergency Department, South West Acute Hospital, Enniskillen, GBR; 2 Surgery, University College Hospital, London, GBR; 3 Histopathology/Cellular Pathology, Mid and South Essex NHS Foundation Trust, Basildon, GBR; 4 Histopathology/Cellular pathology, Basildon and Thurrock University Hospitals NHS Foundation Trust, Basildon, GBR; 5 Radiology, Basildon and Thurrock University Hospitals NHS Foundation Trust, Basildon, GBR; 6 General and Breast Surgery, Mid and South Essex NHS Foundation Trust, Basildon, GBR; 7 General & Breast Surgery, Basildon and Thurrock University Hospitals NHS Foundation Trust, Basildon, GBR; 8 General & Breast Surgery, Anglia Ruskin University, Chelmsford, GBR

**Keywords:** oophorectomy, aromatase inhibitor, tamoxifen, progesterone, estrogen receptor, ovarian metastasis, breast lobular carcinoma

## Abstract

Globally, breast cancer is the most frequently diagnosed malignancy among women; it is also one of the leading causes of cancer mortality among females. The most common sites for metastases are the lungs, bones, liver, and brain. Breast cancer is recognized as one of the most common primary sites of metastatic lesions in the ovaries and is often associated with multiple extra-ovarian metastases. Here, we report a case of occult breast cancer metastases to the ovaries with a down-regulated hormonal immunohistochemistry profile after endocrine therapy, encountered incidentally after oophorectomy.

## Introduction

Globally, the incidence of female breast cancer is 24.2% (i.e., one in four newly diagnosed female cancer cases is breast cancer). It is recognised as the leading cause of cancer death among females (15.0%) [1]. Approximately 70-80% of all breast cancers are estrogen receptor (ER)-positive, implying that hormonal manipulation therapy has a role in the treatment of such cases; nevertheless, therapeutic resistance is not uncommon [2]. ER expression is known to be down-regulated after the use of ER blockade [3].

## Case presentation

**Figure 1:**
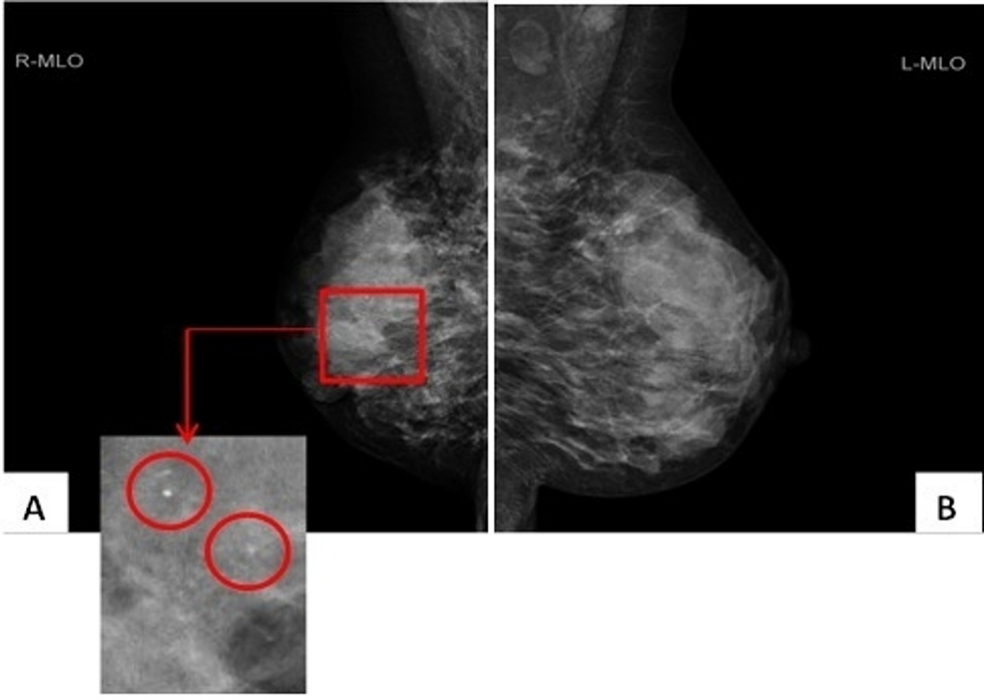
Mammogram: MLO views. (A) The magnified section indicates widespread microcalcification in the right breast seen in the red circled regions, with retracted skin. (B) The left breast appears larger. MLO: medio-lateral oblique

**Figure 2:**
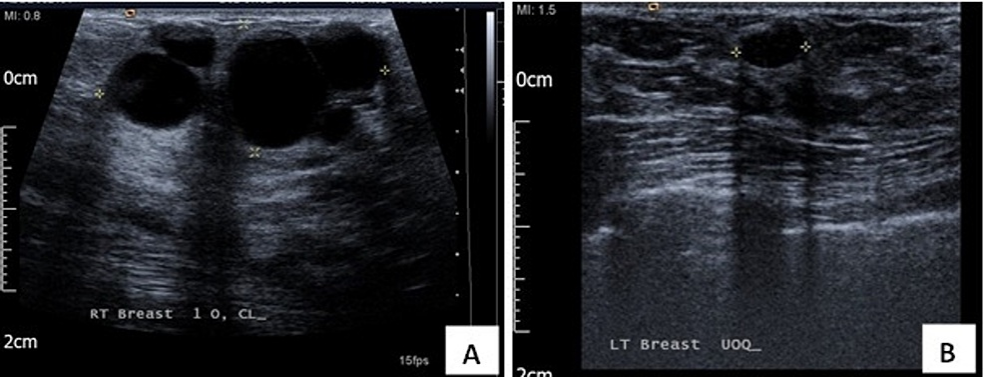
Ultrasound images. (A) Right breast: the entire breast displays an area of irregular abnormality. (B) Left breast: a 7 mm indeterminate lesion in the UOQ area. UOQ: upper outer quadrant

**Figure 3:**
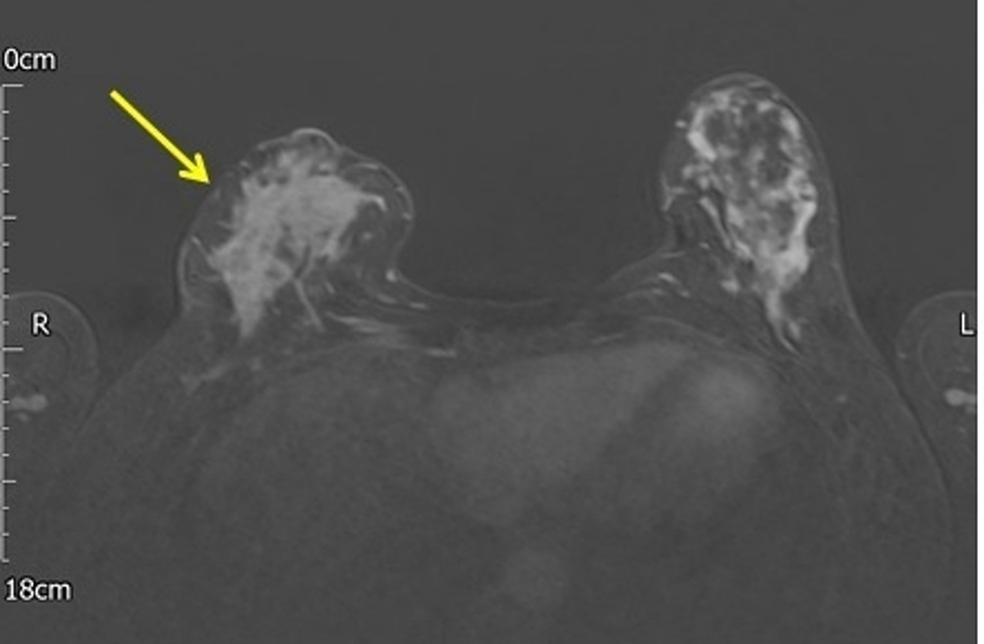
MRI of the breast showing large areas of enhancement involving the entire right breast (yellow arrow). MRI: magnetic resonance imaging

**Figure 4:**
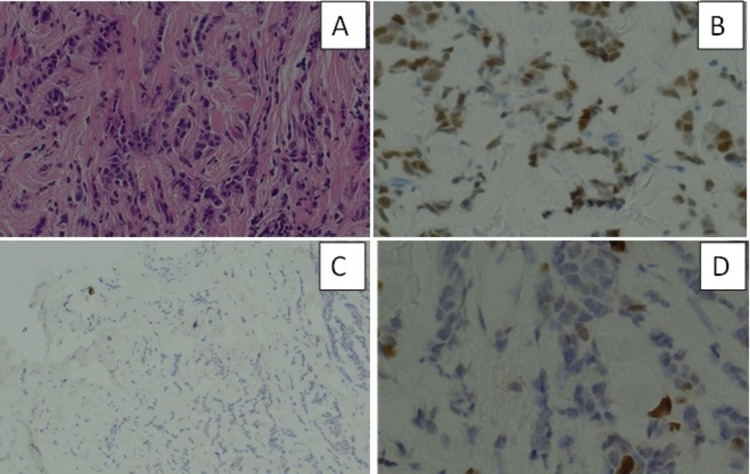
Right breast core biopsy (H&E, 20×). The tumour cells have abundant cytoplasm and some are arranged in a single file (India file) pattern, morphologically consistent with lobular carcinoma. (B) Estrogen receptors staining seven out of eight (40×). (C) E-cadherin immunostaining is negative, consistent with lobular carcinoma (10×). (D) Ki-67 immunostaining is positive in 10% of cells (40×). H&E: hematoxylin and eosin

**Figure 5:**
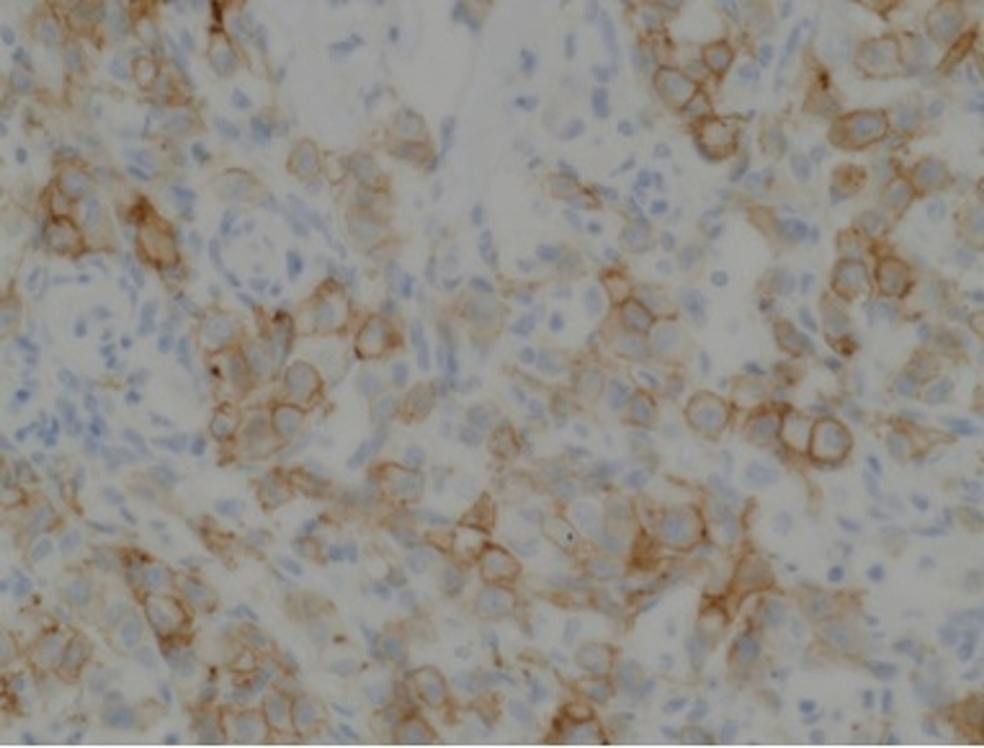
HER-2 IHC (2+ borderline). HER-2: human epidermal growth factor receptor 2; IHC: immunohistochemistry

**Figure 6:**
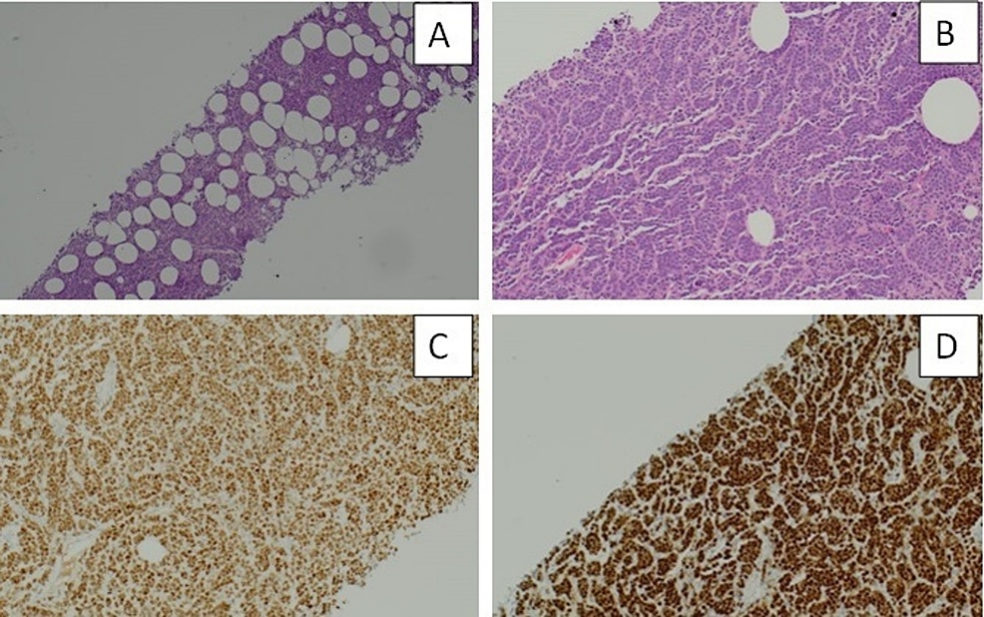
(A) Core biopsy of the right axillary lymph node shows metastasis (H&E, 4×). (B) Core biopsy of the right axillary lymph node shows metastasis (H&E, 10×). (C) ER-positive in the axillary metastasis (10×). (D) PR-positive in the axillary metastasis (10×). H&E: hematoxylin and eosin; ER: estrogen receptor; PR: progesterone receptor

A 42-year-old female presented with enlarged lymphadenopathy and a clinically suspicious painful lump in the right breast. The patient reported no family history of breast or ovarian cancer. The mammogram revealed diffusely spread microcalcification in the right breast (Figure 1). Breast ultrasound showed diffuse hypoechoic abnormality occupying the entire right breast, with several abnormal ipsilateral axillary lymph nodes (Figure 2). In addition, there was a 7 mm indeterminate lesion in the left breast upper outer quadrant (UOQ) area (Figure 2). A breast magnetic resonance imaging (MRI) confirmed these radiological findings (Figure 3). The bilateral breast core biopsy was consistent with grade I invasive lobular carcinoma with ER8, PR8, and Ki-67 of 10% (Figure 4). The human epidermal growth factor receptor 2 (HER2) was borderline (Figure 5). However, fluorescence in situ hybridization (FISH) was not amplified, and the right axillary lymph node biopsy showed metastatic disease (Figure 6).

**Figure 7:**
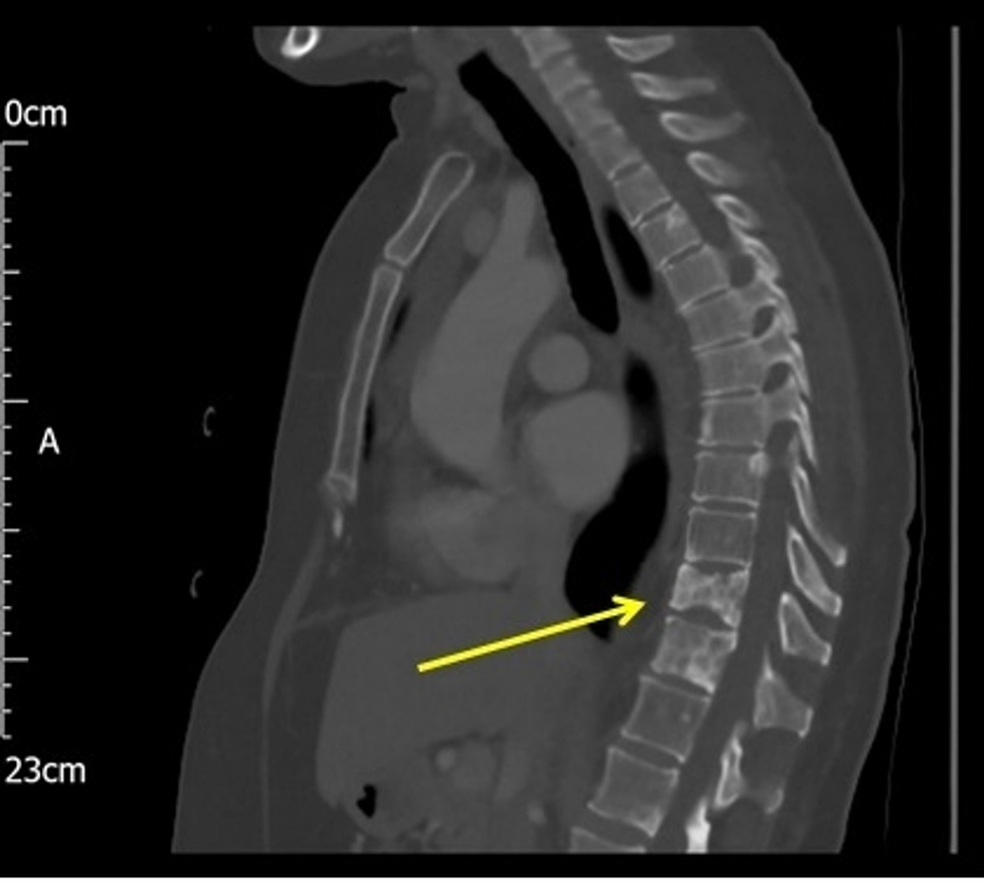
CT chest: sagittal view with multiple lytic and sclerotic lesions in the thoracic spine (yellow arrows). CT: computed tomography

**Figure 8:**
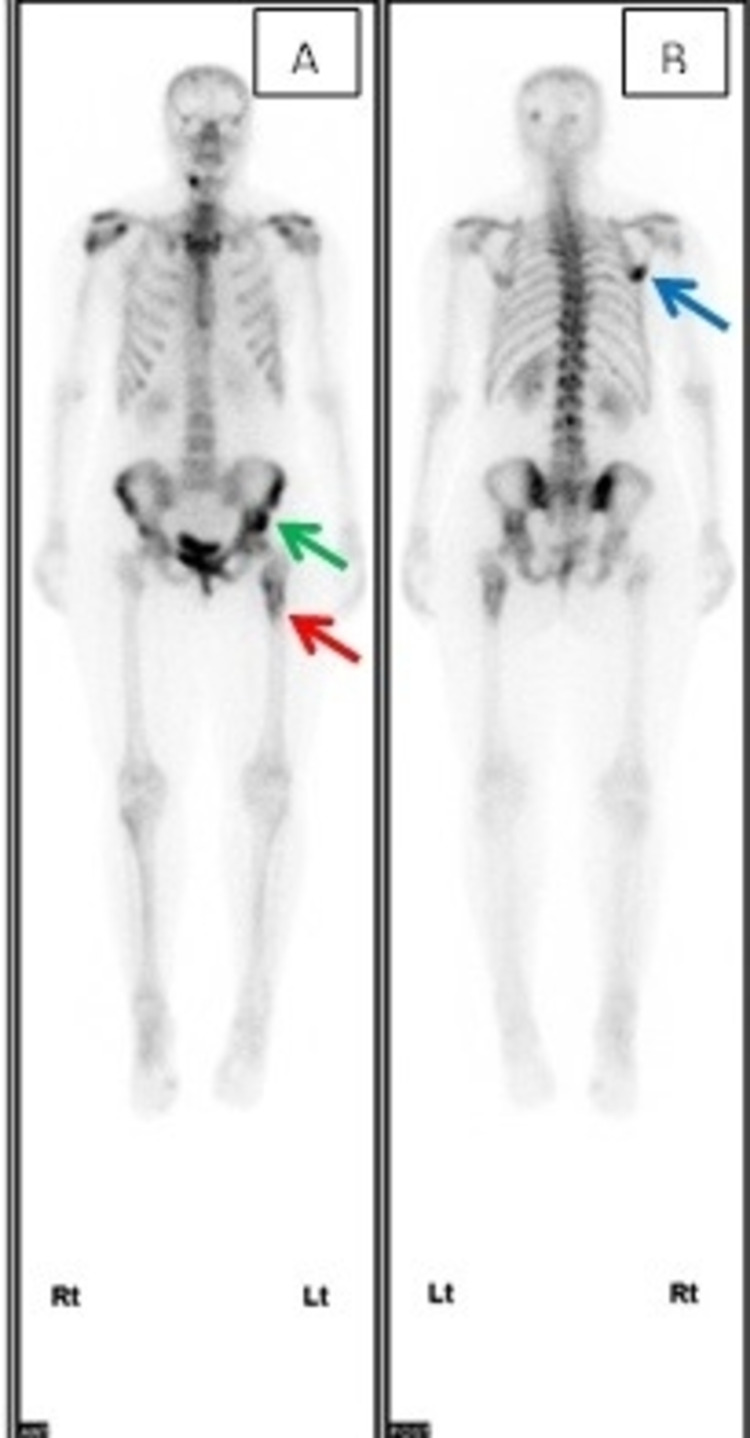
Whole-body bone scan showed increased uptake in (A) the left side of the pelvis (red arrow) and upper left femur (green arrow). (B) Right scapula (blue arrow) lesions are likely to be metastatic.

The staging computed tomography (CT) revealed prominent supraclavicular, bilateral axillary, and para-aortic lymph nodes in addition to multiple lytic and sclerotic lesions in the thoracic spine and pelvic bones (Figure 7). A whole-body bone scan showed increased uptake in the left side of the pelvis, upper left femur, right scapula, skull, and ribs, suggestive of metastasis (Figure 8), and an MRI showed lytic lesions in the surgical neck of both femurs. A multidisciplinary team advised hormonal manipulation using tamoxifen and bisphosphonate (zoledronic acid).

In addition, the patient underwent palliative radiotherapy to the left proximal femur and pelvis. After nine months and due to disease progression, her treatment was switched to letrozole and Zoladex. The treatment goal for metastatic breast cancer in this patient was to maintain a reasonable quality of life.

Due to stable disease and to avoid the side effects of Zoladex, bilateral salpingo-oophorectomy was advised and performed. The post-operative histology showed widespread infiltration by malignant cells involving both ovaries. Immunohistochemistry was positive for mouse monoclonal anti-cytokeratin, pan antibody (MNF116), Cam5.2, cytokeratin 7 (CK7), gross cystic disease fluid protein 15 (GCDFP-15), and GATA binding protein 3 (GATA-3); however, it was negative for ER, progesterone receptor (PR), E-cadherin, CK20, cancer antigen 125 (CA125), and thyroid transcription factor-1 (TTF-1). HER2 was borderline and FISH was not amplified. The findings confirm the diagnosis of metastatic pleomorphic lobular carcinoma originating from the breast.

## Discussion

Breast cancer is the most commonly encountered cancer in women and metastasises mainly to the lungs, bones, liver, and brain, but less frequently to the stomach, rectum, spleen, thyroid, adrenals, kidneys, vagina, uterus, and ovaries [4]. The most common cause of breast cancer-related mortality is metastasis to distant organs. Primary ovarian malignancy is recognised as the fifth most common cancer in females and the leading cause of gynaecological malignancy mortality in females [5]. Ovaries may also harbour metastatic cancer from other remote sites such as the stomach, breast, endometrium, colon, and appendix [6]. Metastatic lesions in the ovaries account for approximately 10% of all ovarian malignancies; 60% originate from extra-genital primaries, with the stomach being the leading source, followed by the breast. There are certain primary breast cancer clinicopathologic criteria that are thought to be related to a higher risk of ovarian metastasis from premenopausal breast cancer. Invasive lobular carcinoma subtype has a threefold higher tendency to metastasise to ovaries and peritoneum than invasive carcinoma of non-specific type (IC-NST) (Table 1). However, because of higher rates of IC-NST among all morphological subtypes of breast carcinomas, there are more IC-NSTs encountered in ovarian metastases from breast cancer. Other disease criteria associated with ovarian metastases include ER-positive disease, node-positive disease, inflammatory cancer, large tumour size, and high histological grade [7]. The proliferation index Ki-67 is used as a prognostic and predictive tool in early breast cancer management [8]. It has been reported that Ki-67 higher than 15% is associated with a higher incidence of metastasis and recurrence; however, this is not specific to ovarian metastasis [9].

**Table 1:** Factors associated with an increased rate of ovarian metastases in breast cancer patients.

Criteria
Age group around 50s
Premenopausal
Invasive lobular carcinoma
Large tumour size
High histological grade
Non-luminal A subtype
Estrogen receptor-positive
Node-positive disease
Inflammatory cancer

**Figure 9:**
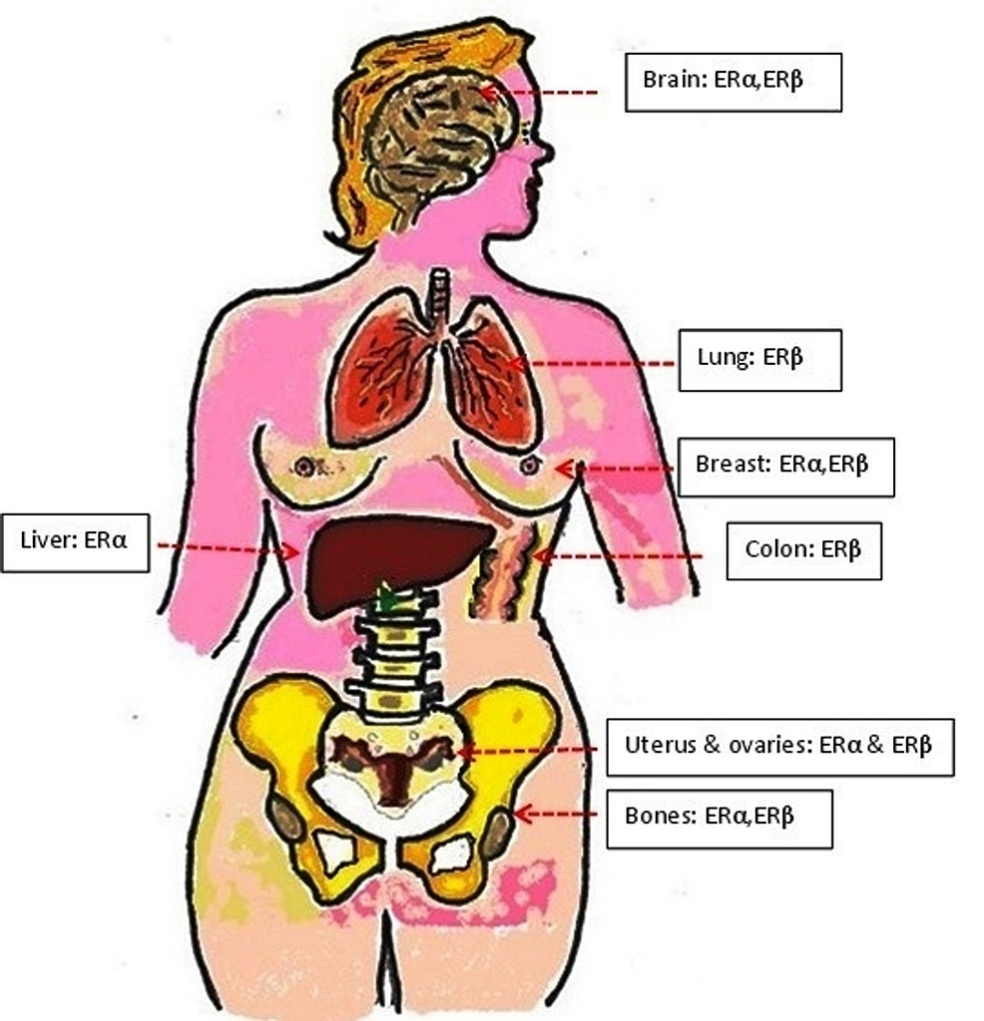
The distribution of ER subtypes in the human body. ER: estrogen receptor Illustration created by Abdalla Saad Abdalla Al-Zawi.

Ovarian metastases from primary breast cancer are repeatedly asymptomatic and often diagnosed as an incidental finding during follow-up imaging or after therapeutic oophorectomy; moreover, they commonly manifest as bilateral pathology [7]. Breast cancer metastases to the ovaries seldom occur as an isolated lesion. Furthermore, invasive lobular carcinoma is more commonly associated with the multi-metastatic phenomenon (25%), wherein the incidence of invasive ductal carcinoma is only 16%. George Beatson in 1896 reported the useful effects of oophorectomy in two female patients diagnosed with inoperable advanced breast cancer. This opened the research gates wide for hormonal blockade breast cancer management [10]. In 1936, Professor Antoine Lacassagne postulated that if breast cancer is connected to a particular inherited sensitivity to estrogen, then the disease may potentially be treated by a therapeutic antagonist to estrogen [11]. Once the relationship between ovarian female hormones and breast malignancy was identified, hormonal manipulation therapy was introduced and implemented as an integral part of the adjuvant treatment component for breast cancer patients with the hormone-dependent disease. The door was opened for scientific research on chemoprevention modalities for breast cancer risk reduction [10]. Lifetime exposure to estrogen and genetic factors play an essential role in breast cancer development. High levels of endogenous estrogen are associated with a higher risk for breast cancer in postmenopausal women, and studies have shown that anti-estrogens decrease the incidence of breast cancer incidence; this also applies to oophorectomy. The mechanism of the estrogen biological action is mediated by two receptor subtypes, ERα and ERβ. Both receptor subtypes are expressed in different cells and both mediate different crucial physiological functions in many organ systems, such as the cardiovascular, musculoskeletal, reproductive, skeletal, and central nervous systems. Estrogen also acts on specific tissues, such as the breast and prostate, as well as affects pregnancy and lactation (Figure 9).

**Figure 10:**
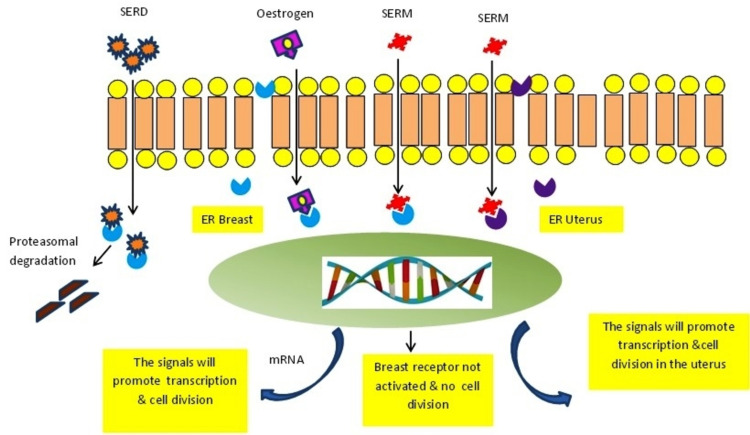
Mechanism of action of estrogen, SERM, and SERD. Estrogen attaches to ER and the produced signals promote transcription and cell division. SERM binds specifically to ER, blocking the estrogen-mediated growth stimuli in breast tumour cells; however the estrogen–SERM complex has a stimulatory effect on uterine cells. ER: estrogen receptor; SERM: selective estrogen receptor modulators; SERD: selective estrogen receptor down-regulator Illustration created by Abdalla Saad Abdalla Al-Zawi.

**Figure 11:**
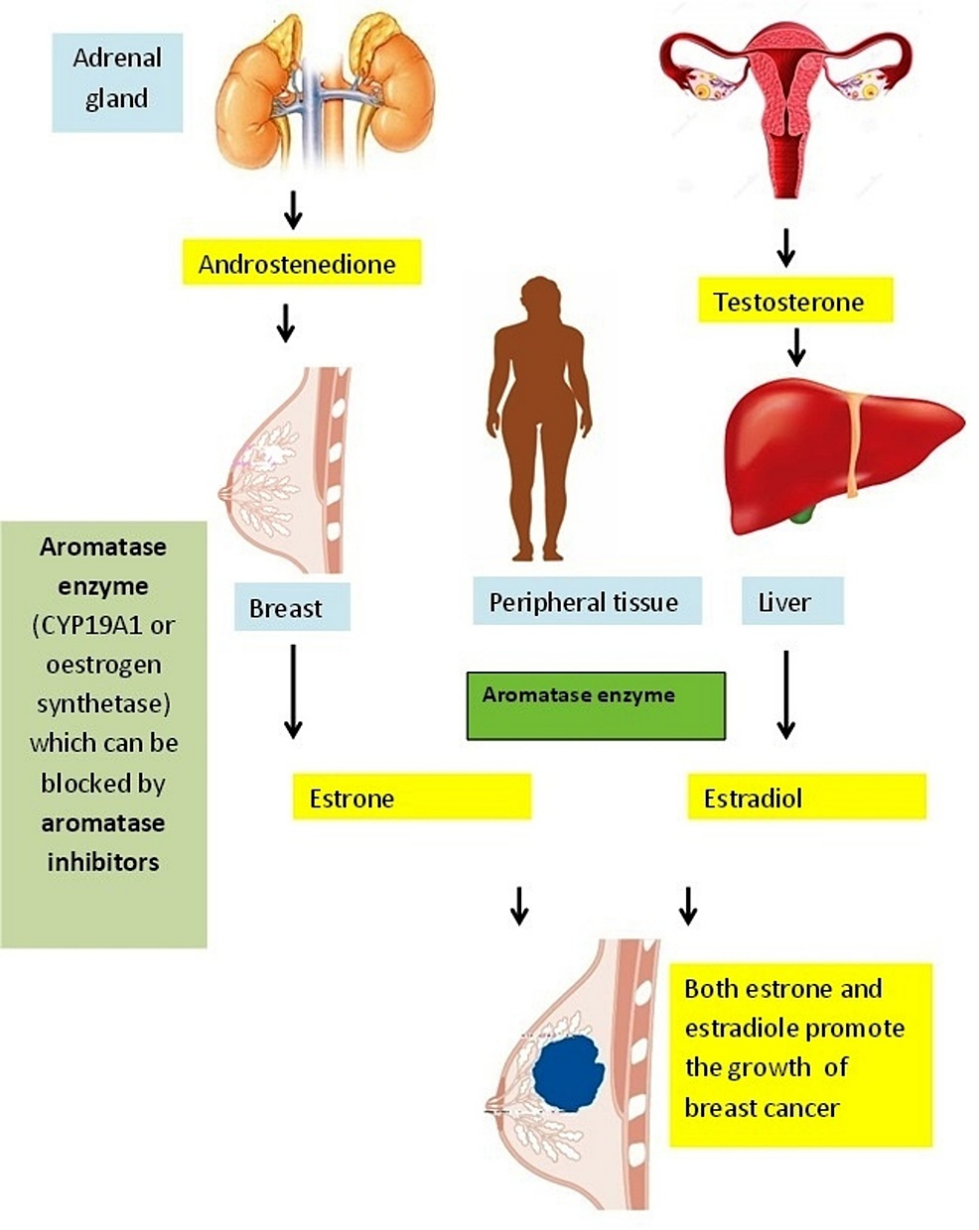
Mechanism of action of aromatase inhibitors which are mainly used in breast cancer treatment in postmenopausal women. The conversion of androstenedione to estrone in the presence of aromatase enzyme occurs in the breast and other tissues. With the help of FSH, androgens (predominantly androstenedione and testosterone) are converted to estrogen by the action of CYP19 A1 (aromatase enzyme). Aromatase inhibitor drugs can block this conversion, leading to lower estrogen hormone available to stimulate breast tumour growth. FSH: follicle-stimulating hormone Illustration created by Abdalla Saad Abdalla Al-Zawi.

ERα subtype or NR3A1 (nuclear receptor subfamily 3, group A, member 1) is expressed mostly in the breasts, thecal ovarian cells, uterus, bone, liver, and fat, as well as in male reproductive organs (mainly prostate stroma, testes, and epididymis). ERβ or NR3A2 (nuclear receptor subfamily 3, group A, member 2) is present commonly in the ovarian granulosa cells, urinary bladder, prostate epithelium, brain, lung, colon, adipose tissue, and blood monocytes and tissue macrophages [12]. Endocrine therapy in the form of anti-estrogen agents is used as an adjuvant treatment of breast cancer. This therapy results in a significant reduction of cancer recurrence and improves the overall survival rate. These drugs include (selective estrogen receptor modulators (SERMs) such as tamoxifen, which binds specifically to ER, blocking the estrogen-mediated growth stimuli in breast tumour cells. Despite its antagonist action in the breast, tamoxifen is associated with a four-fold higher risk of endometrial carcinoma due to its estrogen agonist effect on the uterine cells. This is the reason for the limited use of tamoxifen in postmenopausal breast cancer (Figure 10). Raloxifene, another SERM, is used in the treatment of ER-positive breast cancer without additional risk. Selective estrogen receptor down-regulator (SERD) is an ER-competitive agonist. Fulvestrant is the only SERD used in current medical practice; it degrades ER, preventing its signalling [2]. The other hormonal manipulator drugs, class I aromatase inhibitors (AIs), turn down the endogenous estrogen hormone synthesis in peripheral tissues (Figure 11) and within the tumour cells by inhibiting the enzyme aromatase action. The enzyme aromatase catalyses the conversion of androgenic precursors (testosterone and androstenedione) to estrogens (estradiol and estrone) [2].

There are two types of AIs. Type I AIs are steroidal and include exemestane, a modified androstenedione. Type II, non-steroidal AIs include letrozole and anastrozole. The steroidal (type I) AIs bind to the enzyme aromatase, irreversibly inactivating it. Type II AIs bind to the active aromatase site, inhibiting it; however, unlike type I, this inhibition is reversible. The AIs are used as first-line adjuvant endocrine treatment options for postmenopausal patients. In early and metastatic hormone-positive breast cancer, AIs are considered for some premenopausal women, in addition to ovarian suppression by gonadotropin-releasing hormone agonists [2]. Previous studies have reported that ER expression alteration can occur after a period of treatment with tamoxifen, and ER-positive tumours, which initially responded to the hormonal blockade, would later become refractory to treatment (having acquired resistance). The reduction in ER expression occurs on the plasma membrane, including the alternative G-protein coupled receptor (GPR-30) and ERɑ.

Tamoxifen-resistant cells use different pathways to stimulate cell proliferation in the absence of genomic estrogen signalling as the inflammation-associated transcription factor nuclear factor-kappa B (NF-κB), epidermal growth factor (EGF), and insulin-like growth factor (IGF-1) pathway [13]. A decrease in response to AIs is either primary/de novo resistance or secondary/acquired resistance. If tumour relapse occurs within the first 24 months of adjuvant endocrine therapy, the resistance is regarded as primary. The resistance is classified as secondary/acquired resistance if the relapse occurs while the patient is on adjuvant endocrine therapy, but the treatment period is beyond the first two years; if the relapse occurs within 12 months of finishing adjuvant endocrine therapy; or if a disease progression has been noticed after six months of initiating endocrine therapy for metastatic breast [14]. Few patients develop acquired resistance to hormonal blockade treatment due to the loss of expression to ERɑ receptors or because the molecular alteration of ER influences the ligand-binding domain. In contrast, PR expression loss occurs more frequently. Alteration of receptor expression status has been linked to a higher level of growth factor signalling and increased resistance to hormonal manipulation therapy, transforming the disease into more clinically aggressive tumours and leading to worse patient outcomes. There is a real challenge in scenarios, such as in this case, where the differentiation between the primary and metastatic ovarian malignancy is difficult, especially in cases of *BRCA* gene mutation or Lynch II syndrome, wherein both primary breast cancer and primary ovarian malignancy may coexist. The immunohistochemistry panel is crucial in differentiating metastatic breast cancer to the ovaries from primary ovarian cancers. Cytokeratin MNF116 is a broad-spectrum anti-cytokeratin epithelial marker reacting with some keratins as CK5, 6, 8, 17, and 19. It is a useful aid in the detection of malignancies of epithelial origin such as adenocarcinoma. In this case, it was positive in the ovarian tumour. Cam5.2 is an antibody marker often used to detect CK8 and, to a lesser extent, CK7, and confirms the epithelial origin of the tissue as breast cancer; this marker was positive in the ovarian specimen in our case. CK7 is a known type II keratin marker found in the simple non-keratinizing epithelium, seen in the breast, lung, and gut tissue. In the presented case, it was positive in the ovarian sample. The CK20 (46-kDa acid protein) presents in the epithelium of the gut, bladder, pancreas, and biliary tract, as well as in Merkel cells, and is essentially negative in breast cancer and ovarian cancer; however, it may express some positivity in lung adenocarcinoma. CK7-positive and CK20-negative immunohistochemistry profile is seen in breast cancer, pulmonary adenocarcinomas, and thyroid cancer. Our case showed CK7-positive/CK20-negative pattern in the ovarian specimen. GCDFP 15 is found in the breast tissue; it is a useful specific immunohistochemistry marker for breast neoplasms and was negative in the ovarian tumour specimen. GATA-3 is a nuclear marker protein expressed in many epithelial neoplasms such as breast and urothelial tumours and phaeochromocytoma [15]. It plays an important role in the detection of metastases; in our case, it was positive in the ovarian tumour. CA-125 is a protein encoded by the *MUC16* gene, found in the female reproductive tract epithelium, respiratory tract, and cornea. It is used as a tumour marker in ovarian cancer with 79% sensitivity; however, it is not specific as it can be elevated in other neoplasms and inflammatory conditions. It is an important marker to differentiate primary ovarian carcinoma from metastatic breast cancer to the ovary [16]. CA-125 was not expressed in the ovarian tumour in our case. TTF-1 is a nuclear protein that regulates the transcription activity of thyroid and lung proteins and is used as tumour markers in the neoplasms originating in their tissues. TFF-1 expression can be encountered in ovarian cancers [17]; in our case, it was negative. ERs are expressed in approximately 70-80% of female breast neoplasms, while PR expression is reported to be positive in 63% of breast cancers [18]. ER and PR may also be positive in ovarian cancer in up to 90% and 64%, respectively [19]; neither marker was detected in our case. The calcium-dependent protein E-cadherin is expressed normally in normal breast epithelial tissue. Moreover, it functions as an essential component of epithelial cell-to-cell adhesion and epithelial-to-mesenchymal transition. A lack of E-cadherin expression confirms breast invasive lobular carcinoma. E-cadherin was not expressed in the ovarian tumour in our patient. In cases of a higher risk of genetic predisposition to breast and ovarian cancers, and because of the potential impact of the adjuvant hormonal manipulation therapy of breast cancer on the uterus, regular gynecologic examinations and pelvic ultrasound are recommended to enable early detection of ovarian tumours, in addition to routine mammogram surveillance of breast cancer after primary treatment [20]. In isolated ovarian metastatic disease, metastasectomy is indicated for diagnostic and therapeutic purposes, which can be done by simple laparoscopic or open bilateral salpingo-oophorectomy [7]. As mentioned earlier, metastatic breast cancer to the ovaries is commonly a component of disseminated metastatic disease; however, removal of the ovaries in such cases by cytoreductive surgery (oophorectomy or debulking technique to remove all pelvic macroscopic lesions) is recommended for tissue diagnosis, removal of the tumour, and removal of the estrogen source. This is regarded as a part of endocrine therapy in premenopausal hormone receptor-positive disease. Targeted therapy and systemic therapy with hormonal manipulation, chemotherapy, and HER2 blockade are indicated to control the other disseminated disease foci and improve the patient’s quality of life [7,20].

## Conclusions

This case presents an example of ER down-regulation phenomenon after hormonal manipulation treatment in metastatic breast cancer. This may be associated with disease progression, as seen in our case, and such a scenario can severely impact further treatment choices. Therefore, it is advisable to re-check the receptor status in metastatic breast cancer in a patient after a period of treatment. Oophorectomy should be considered as a valuable palliative treatment modality for metastatic breast cancer to deprive the body of estrogen hormones and to remove a potential site for metastases.
